# Investigating a caregiver-assisted social skills group programme for primary and early high school-aged children with acquired brain injury or cerebral palsy: protocol for a pilot mixed-methods, two-group randomised trial of PEERS Plus

**DOI:** 10.1136/bmjopen-2024-095354

**Published:** 2025-01-04

**Authors:** Bianca Thompson, Rose Gilmore, Nicola Hilton, Jacqui Barfoot, Christine T Moody, Koa Whittingham, Afroz Keramat, Roslyn N Boyd, Leanne Sakzewski

**Affiliations:** 1 Faculty of Medicine, The University of Queensland Child Health Research Centre, South Brisbane, Queensland, Australia; 2 University of Queensland, South Brisbane, Queensland, Australia; 3 Queensland Paediatric Rehabilitation Centre, Brisbane, Queensland, Australia; 4 Queensland Paediatric Rehabilitation Service, Children's Health Queensland Hospital and Health Service, Brisbane, Queensland, Australia; 5 Queensland Cerebral Palsy and Rehabilitation Research Centre, The University of Queensland, South Brisbane, Queensland, Australia; 6 UCLA Jane and Terry Semel Institute for Neuroscience and Human Behavior, Los Angeles, California, USA; 7 Queensland Cerebral Palsy Research Centre, The University of Queensland, Brisbane, Queensland, Australia; 8 The University of Queensland, Saint Lucia, Queensland, Australia; 9 Children’s Allied Health Research, Queensland Health Centre for Children's Health Research, South Brisbane, Queensland, Australia

**Keywords:** Community child health, Child, Brain Injuries, Social Interaction

## Abstract

**ABSTRACT:**

**Introduction:**

Reaching social milestones is an important goal of childhood. Children with acquired brain injury (ABI) and cerebral palsy (CP) frequently experience challenges with social functioning and participation. The Programme for the Education and Enrichment of Relational Skills (PEERS) is a group-based social skills programme for adolescents. This study will compare an adapted PEERS programme with usual care in a pilot randomised waitlist-controlled trial for primary and early high school-aged children with brain injuries.

**Methods and analysis:**

This single-centre study will be conducted at the Queensland Cerebral Palsy and Rehabilitation Research Centre at the Centre for Children’s Health Research in Brisbane, Australia. Thirty-two school-aged children (grades 3+; 8–13 years) with an ABI or CP and their caregiver(s) will be recruited and randomly assigned to either 12 week PEERS Plus or waitlist usual care. The waitlist group will then participate in PEERS Plus after the 3 month retention time point. The primary outcome will measure individualised social participation goals on the Canadian Occupational Performance Measure immediately postintervention at the primary endpoint (12 weeks). Secondary outcomes include the Social Skills Improvement System Social-Emotional Learning Edition Rating Forms and Quality of Play Questionnaire immediately postintervention, 12 weeks postintervention (26 weeks postbaseline), 36 weeks postintervention (52 weeks postbaseline) for retention. Following completion of the PEERS Plus programme, semistructured focus group interviews will be conducted separately with caregivers and children to explore the lived experience of PEERS. Interpretive description will be used to identify patterns and themes related to participants’ experiences. Analyses will follow standard principles for randomised controlled trials using two-group comparisons on all participants on an intention-to-treat basis. Comparisons between groups for primary and secondary outcomes will be conducted using regression models. This study will estimate the unit costs of providing PEERS Plus at different levels of public health facilities in Australia.

**Ethics and dissemination:**

This study has been approved by the Medical Research Ethics Committee of The University of Queensland (2022/HE002031) and the Children’s Health Queensland Hospital and Health Service Human Research Ethics Committee (HREC/22/QCHQ/87450). Recruitment and participant informed consent process will be completed in accordance with institutional ethic procedures. Dissemination plans include peer-review publication of study results, presentations, and instructional workshops at national and international conferences.

**Trial registration number:**

ACTRN12623000515695.

**Strengths and limitations of this study**

- This study addresses a recognised gap in the continuum of care for primary and early high school-aged children with brain injury.
- This trial will explore the perspectives and experiences of school-aged children (grades 3+; 8–13 years) and caregivers who participate in the Programme for the Education and Enrichment of Relational Skills (PEERS) Plus programme.
- Inclusion of a 3 and 9 month follow-up to assess maintenance of treatment gains.
- Due to the nature of the intervention, participants and the intervention providers will not be blinded to treatment allocation.

## Introduction

 Children with acquired brain injury (ABI) and cerebral palsy (CP) are at an increased risk of social functioning difficulties and social participation restrictions when compared with their typically developing peers.^1^,^4^ It is estimated that 10%–15% of children in the general population experience social functioning impairments,^5 6^ between 23% and 50% of children with ABI,^1 7 8^ and 33%– 45% of children with CP experience social difficulties.^3 9^

Defined as ‘the interaction of an individual with their environment and the ability to fulfil meaningful roles within their environment’ (eg, social activities, community participation and relationships with family) (Bosc, p.63),^10^ social functioning is an overarching construct encompassing the integration of social, behavioural and cognitive skills.^11^ Performance in social functioning develops from early childhood to adolescence; however, for children with ABI and CP, there are often barriers and restrictions to attaining social milestones across the school years.^2 11^

During the primary school years, reaching social milestones, including the ability to interact aptly with peers, is an important goal. Children who fail to attain desired and required skills are at an increased risk of adverse developmental outcomes in later school years, including difficulties adjusting after school transitions, poor academic achievement, peer rejection, risky behaviour in adolescence and mental health problems.^911^,^13^ Children with ABI and CP are at additional risk of adverse social outcomes, as a higher proportion are socially rejected compared with peers without disability, are considered less popular in a group, have fewer friends and are bullied more often than children without brain injury.^14^,^17^ Additionally, they participate in fewer social activities and are not presented with the same learning opportunities through social experiences.^18^ Unaddressed social functioning difficulties during school years are likely to persist into adulthood, placing individuals at risk for multiple forms of maladjustment problems^19^ and mental health problems.^20 21^

Group social skills interventions (GSSIs) have been developed and tested predominantly with children diagnosed with autism. Meta-analyses of GSSIs for young people with autism have identified improvements in social competence, responsiveness, knowledge and skills associated with participation.^22^,^24^ Recently, there has been increased attention testing the efficacy of GSSIs in other age brackets (such as preschoolers with autism) and disability groups, such as children and young people with attention-deficit hyperactivity disorder.^23^,^28^ GSSIs are typically informed by cognitive–behavioural,^29^,^31^ theory of mind^32 33^ and behavioural strategies for teaching social skills centred on social learning theory.^34^ GSSIs aim to teach individuals the skills necessary for success in everyday social interaction.^35^ Programmes frequently offer the innate adaptability and flexibility to be delivered across diverse settings (eg, schools, holiday camps and outpatient clinics) and locations (eg, face-to-face and virtual/telehealth).^23^ Teaching components include but are not limited to role-play and modelling, didactic instruction, behavioural rehearsal, positive reinforcement and homework tasks.^36^ The inclusion and consideration of creative, dramatic and theatrical approaches to facilitate and promote the generalisation of learnt skills to everyday environments have also featured.^37^,^39^ In recent years, more positive outcomes (eg, formation of appropriate peer networks and translation of learnt skills) have been associated with programmes that include caregiver involvement.^40 41^ Specifically, GSSIs that included caregiver groups were deemed more effective, with an associated large effect size (Standardised Mean Difference, SMD −0.91, 95% CI −1.20 to –0.61; Z=6.08), compared with the moderate effect size (SMD −0.63, 95% CI −1.23 to –0.02; Z=2.03) associated with GSSIs without caregiver components.^23^

The most widely researched and internationally recognised GSSI is the Programme for the Education and Enrichment of Relational Skills (PEERS).^24 40^ As an evidence-based caregiver-assisted GSSI, PEERS is associated with social skills improvements and long-term maintenance of gains in social competence 1–5 years following intervention.^2342^,^44^ Various formats of the programme are now publicly available for adolescents and young adults,^40 45^ as well as a more recent extension for preschoolers with ASD, the PEERS for Preschool programme (P4P).^46^,^48^

In its original format, the manualised PEERS programme comprised 14 weekly in-person sessions with adolescents and their caregivers attending co-occurring groups. Utilising principles of cognitive–behavioural therapy (CBT), PEERS integrates a range of teaching approaches and applies teaching methods of didactic instruction (psychoeducation), cognitive strategies, behavioural rehearsal, role-play demonstration and performance feedback within a small group therapy setting.^31 40 45^ Each session focuses on teaching ecologically valid social skills, including associated homework tasks and review, and promoting the generalisation of learnt skills to other settings and environments.^40^ PEERS caregiver sessions directly mirror adolescent sessions; with each session focusing on reviewing the prior week’s homework tasks, sharing what the adolescent group is learning and assigning homework tasks.^40^ Caregiver sessions further offer contemporary tools and strategies to assist caregivers in acting as social coaches for their child in everyday settings. Caregiver involvement is deemed an essential component of this intervention, supporting the generalisability and the sustainability of the outcomes of this intervention in the long term.^2342^,^44^

While GSSIs such as PEERS have been implemented successfully across various settings and populations, evidence for the efficacy of group-based social skills programmes for adolescents and children with ABI and CP is scarce.^24^ To the best of our knowledge, only one randomised controlled trial (RCT) of PEERS with adolescents with ABI and CP has been conducted.^49^ There were no differences between groups on the primary outcome, The Social Skills Improvement System (SSIS)^50^ . For secondary outcomes, adolescents with brain injuries, who completed PEERS compared with a control group, demonstrated improved social knowledge measured on the Test of Adolescent Social Skills Knowledge (TASSK) (TASSK, mean difference (MD)=6.8, 95% CI 4.8 to 8.8; p<0.001), which were maintained at the 26 week retention time point (MD=8.1, 95% CI 6.0 to 10.2; p<0.001). The programme was further associated with a significant increase in parent-reported invited get-togethers at 26 weeks (incidence rate ratio 4.0, 95% CI 1.0 to 16.0; p=0.05).^49^

### Aims and hypotheses

#### Broad aim

PEERS Plus is a novel programme developed for school-aged children with ABI and CP who experience difficulties in social functioning. Adapted from The Programme for the Education and Enrichment of Relational Skills (PEERS), PEERS Plus includes didactic instruction, behavioural rehearsal, positive reinforcement, role-play, creative arts activities, interactive games and modelling to promote skill generalisation. The aim of this study is to pilot test an adapted PEERS programme ‘PEERS Plus’ with school-aged children (grades 3+; 8–13 years with ABI or CP) to improve social functioning and social participation in a parallel waitlist RCT. Another key objective is to assess the costs associated with implementing the PEERS Plus programme, offering decision-makers an estimate of the resources required to put this evidence-based programme into practice. Our secondary aim is to explore the experiences of PEERS Plus for children and caregivers who partake.

#### Primary hypotheses

For school-aged children (grades 3+; 8–13 years) with ABI or CP, PEERS Plus will be more effective than a waitlist control group receiving usual care to:

Achieve significantly greater self-reported performance of social participation goals as measured on the Canadian Occupational Performance Measure (COPM),^51^ by two points or greater.

#### Secondary hypotheses

For school-aged children (grades 3+; 8–13 years) with ABI or CP, PEERS Plus compared with a waitlist control group receiving usual care will result in:

1. Greater self-perceived satisfaction with social participation goals (COPM).^51^
2. Greater caregiver-reported social skills and competence (Social Skills Improvement System Social-Emotional Learning Edition Rating Forms (SSIS-SEL)).^52^
3. Greater parent-reported frequency of play dates with friends (Quality of Play Questionnaire (QPQ)).^53^
4. Decreased self-reported episodes of bullying and victimisation (The Personal Experiences Checklist (PECK)).^54^
5. Increased social knowledge (The Test of Child Social Skills Knowledge (TCSSK)).
6. Increased caregiver-reported knowledge, skills and confidence in coaching their child (caregiver social coaching questionnaire for PEERS).
7. Greater self and caregiver-proxy reported quality of life (Kidscreen-27).^55^
8. Decreased parent-reported behavioural and emotional problems (The Strengths and Difficulties Questionnaire (SDQ)).^56^
9. Greater quality-adjusted life years (QALYs) (The Child Health Utility 9D (CHU9D)).^57^

Caregivers and school-aged children will also provide insight into the lived experience of participation in PEERS Plus (including barriers and enablers of participation), perceived changes in social functioning and participation and suggestions for adaptions of the intervention (focus groups).

## Methods and analysis

### Study design

This single-centre pilot study will be conducted at the Queensland Cerebral Palsy and Rehabilitation Research Centre at the Centre for Children’s Health Research in Brisbane, Australia. A pilot design will be employed to examine the efficacy of PEERS Plus, address questions concerning feasibility and acceptability, and fill gaps in knowledge about aspects of the intervention and study conduct.^58^

**Figure 1:**
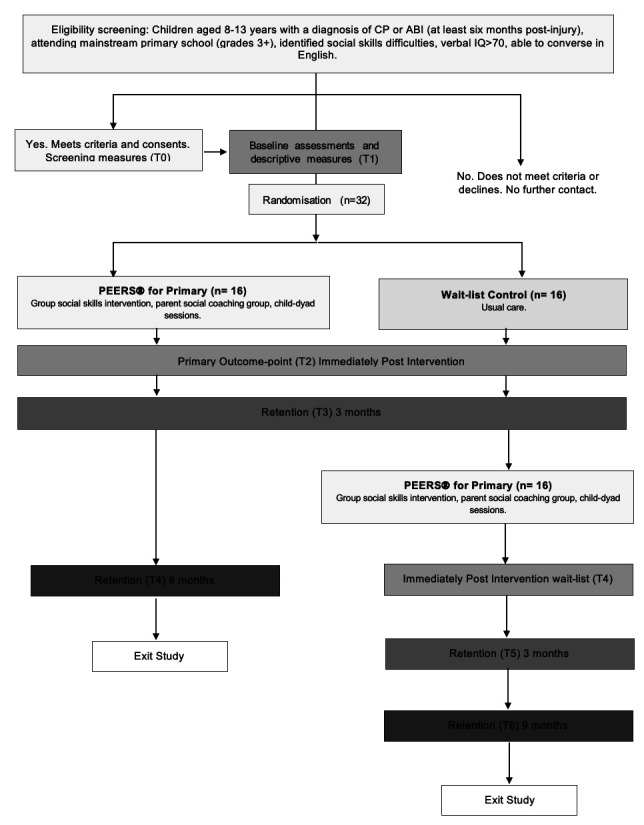
CONSORT study flow diagram. ABI, Children with acquired brain injury; CONSORT, Consolidated Standards of Reporting Trials; CP, cerebral palsy; PEERS, Programme for the Education and Enrichment of Relational Skills.

This study will use a parallel waitlist pilot-randomised two-group, pretest, post-test study (over 12 weeks), inclusive of screening, descriptive measures and consent (T0), baseline assessments (T1), follow-up immediately postintervention at the primary endpoint (T2; 12 weeks), 12 weeks postintervention (T3; 26 weeks postbaseline) and 36 weeks postintervention (T4; 52 weeks postbaseline) for retention (refer to figure 1). The waitlist group will go on to participate in PEERS Plus after the 3 month retention time point.

The intervention group will be compared with a waitlist group receiving care as usual, as the target population receive minimal direct therapy related to social functioning. This approach could be considered more ethical, as all persons involved are provided with the opportunity to experience the intervention.

Immediately after the programme’s completion, children’s and caregivers’ subjective perceptions and experiences of the programme will be captured via focus groups. An interpretive description strategy (ie, an inductive approach inspired by phenomenology, grounded theory, ethnography and naturalistic enquiry)^59^ will be employed to guide the process of capturing patterns and themes related to participants’ experiences. This approach aims to generate knowledge and a contextual understanding that will guide further investigation of social skills interventions for children with brain injuries.

Group video footage of each session will be collected and archived, given the nature of this pilot study. Session recordings may aid in the process of making any necessary adjustments and refinements to the PEERS Plus programme following the completion of this pilot study.

Study design details are reported in accordance with the Standard Protocol Items: Recommendations for Intervention Trials (SPIRIT) statement.^60^ The Consolidated Standards of Reporting Trials (CONSORT) guidelines (figure 1),^61^ and Template for Intervention Description and Replication (TIDier) guideline,^62^ were used to guide the reporting of intervention in this protocol.

### Recruitment

Thirty-two children (grades 3+; aged 8–13) at study entry with ABI or CP living in the Brisbane metropolitan area and surrounds will be recruited. Families with a child meeting eligibility criteria will be identified via referrals from the Queensland Paediatric Rehabilitation Service at the Queensland Children’s Hospital and invited to join the study by the study coordinator via mailout or phone. Recruitment will also occur via word of mouth and social media, that is, posting the study flyer to various social media platforms such as LinkedIn and Facebook.

Following recruitment, eligibility screening will be conducted in accordance with the guidelines outlined in the PEERS for adolescents manual. This includes a 10–15 min telephone screening with the child’s primary caregiver and the study coordinator, who is not involved in the delivery of the programme. During the screening call, the culture of the programme will be described, and the child’s primary caregiver will be informed of the associated risks of participating in the programme. If the caregiver or child is not interested or motivated, they will not proceed to the screening interview or consent process (T0) and will not be enrolled in the study.

Recruitment for this trial commenced in May 2023 following ethical and governance approvals. This study has been approved by the Medical Research Ethics Committee of The University of Queensland (2022/HE002031) and the Children’s Health Queensland Hospital and Health Service Human Research Ethics Committee (HREC/22/QCHQ/87450).

### Inclusion criteria

To meet eligibility for inclusion, children must:

1. Have a diagnosis of ABI (at least 6 months postinjury) or CP.
2. Have difficulties with social functioning as reported by their caregiver.
3. Be motivated to participate in the programme and learn skills to develop friendships (determined through self-report during screening procedures).
4. Attend mainstream school and are in grades 3+ (aged 8–13 years).
5. Have a verbal IQ>70 measured on the Wechsler Abbreviated Scale of Intelligence second Edition (WASI-II).^62^
6. Use speech that is understandable to unfamiliar listeners.
7. Have a caregiver that is willing and able to attend the programme to participate in parent-component of intervention.
8. Have, along with their attending caregiver, basic proficiency in English (eg, understand the content of the sessions, able to complete questionnaires, and participate in group discussions).
9. Be able to commit to the preassessments and post-assessments, 12 weekly sessions and complete homework tasks.

### Exclusion criteria

Children will be excluded if they:

1. Have uncontrolled epilepsy, not controlled by medication.
2. Have severe visual or auditory impairment.
3. Are non-verbal.

### Randomisation

A randomisation schedule will be generated using a computer-generated random number sequence.^2^,^4^ Group allocation was concealed in consecutively numbered, sealed, opaque envelopes, created by personnel not involved in recruitment or delivering the intervention. Envelopes were made available to the assessor after completion of baseline assessments when allocation was revealed to participants and study personnel.

### Blinding

Due to the nature of the intervention, participants and the intervention team will not be blinded to treatment allocation. The primary outcome (COPM) is a self-report measure, and therefore, it is not possible to be blinded. Child–caregiver dyads will complete the COPM with a clinician who will not be blinded to group allocation. At follow-up, the clinicians completing the COPM semistructured interviews and assessments will be blinded to previous scores. All other outcome measures are questionnaire-based and either self or proxy-completed; therefore, blinding is not possible.

### Adverse events and safety

In the former waiting list RCT of PEERS with adolescents with ABI and CP, it was noted that no adverse events occurred.^49^ Thus, due to similarities between studies and the nature of the intervention, we expect a low likelihood of adverse events. If any near misses or adverse events occur, incidents will be documented and reviewed by the principal investigator (PI) (LS) and followed up in accordance with site-specific requirements and institutional procedures. Any moderate or severe adverse events will be reported to the Ethics Committee as per ethical reporting obligations. Clinicians will be available at the end of each session for a brief meeting with each dyad if the child or caregiver wishes to discuss any concerns regarding their ongoing participation in the programme.

### PEERS Plus intervention

The programme and detailed description of the sessions and content are summarised in table 1.

**Table 1:** Overview of sessions and content in PEERS Plus

Dyad sessions	Child sessions	Caregiver sessions
Collaborative goal setting(baseline, T1)		
Problem-solving and coaching social participation goals (approx. weeks 1–2)	(1) Introduction│focusing attention and active listening	Review of all child session topics Question opportunities Peer networking Other:Parenting strategies: Praising Prompting Delivering feedback Reinforcement
	(2) Facial expressions, emotions and body language	
	(3) Trading information and using your voice	
	(4) Two-way conversations	
	(5) Choosing friends and using humour	
	(6) Entering and exiting a conversation	
	(7) Entering and exiting a game	
Midpoint goal review (approx. weeks 4–8)	(8) Being a good sport	
	(9) Get-togethers	
	(10) Handling disagreements	
Endpoint goal review (approx. weeks 11–12)	(11) Teasing, bullying and embarrassing feedback	
	(12) Review of skills and graduation	

PEERS Plus is an adapted version of PEERS specifically developed and tailored for primary and early high school-aged children (grade 3+, aged 8–13 years) with ABI and CP who have difficulty with social functioning. The PEERS Plus adaptation was completed by the first author, BT, and reviewed by five authors: RG, NH, JB, LS and CM. CM, a licenced clinical psychologist, and the Director of Research at the UCLA PEERS Clinic, Semel Institute for Neuroscience and Human Behaviour, where PEERS was developed, ensured that the programme stayed true to PEERS.

PEERS Plus comprises 12 weekly group sessions, with an additional three individual sessions offered to each child–caregiver dyad. Following collaborative goal setting of 2–3 social participation goals (baseline, T1), the first individual child–caregiver dyad session will focus on problem-solving and coaching social participation goals. The further two sessions will be offered around the mid and endpoint of the programme to review the progression of set goals, with the timing of individual sessions being flexible and reflecting each child–caregiver dyad needs.

The 12 weekly child group sessions will consist of up to eight children, each session totalling 90 min, with afternoon tea provided 30 mins prior to the start of each session. Each week a concurrent caregiver group will run alongside the child session in a separate room. Two clinicians per group will deliver the manualised programme each week, with at least one clinician certified in administering the original manualised PEERS for adolescents programme and all clinicians trained in PEERS Plus. Clinicians not trained in the programme will attend a formal training session prior to the commencement of the programme; they will further refer to the intervention manual and receive guidance from certified clinicians to assist in running the groups. Debriefing time will be made available to all clinicians before and after each session, with formal debriefing times offered during bi-weekly team meetings throughout the intervention.

Caregiver sessions will run for 90 min with an allocated 30 min before each session for ‘peer networking’ (ie, informal group discussion opportunities). During the last 10–15 min of each session, caregivers will be invited to join the child group for the allocation of homework tasks. On an as-need basis, following the allocation of homework tasks, clinicians will be available to discuss any questions or concerns with each child–caregiver dyad.

The content of PEERS Plus child sessions is adapted from the original PEERS manual,^40^ with additional sessions (sessions 1, 2, 7) and content incorporated to address the needs of a younger age bracket (eg, session 7: entering and exiting a game) and themes outlined in the brain injury literature (eg, communication, emotion recognition and social problem-solving).^63^ Further adjustments to the programme in response to consumer feedback include three additional individual sessions to provide individual support.^64^ Revised ‘home missions’ (ie, homework tasks) are allocated each week to accompany the session and support the generalisability of the intervention.

Programme components vary between sessions, including but not limited to didactic instruction, behavioural rehearsal, positive reinforcement, role-play opportunities, interactive games and modelling opportunities to promote the generalisation of learnt skills to everyday environments. Sociodramatic (eg, sociodramatic play) and creative arts-based activities (eg, theatre games and art activities) were also included as an adaptation to the PEERS programme to maximise engagement and motivation of a younger age group and allow for additional behavioural rehearsal and social coaching opportunities in an engaging, targeted and age-appropriate manner.

### Tailoring and audit trail

Minor programme tailoring is anticipated due to the nature of this pilot study. Clinicians and project team members will meet weekly after each session to discuss and record any content adjustments required to ensure the suitability of the programme. Any adjustments and modifications made during the 12 weeks will be recorded in the PEERS Plus manual. It is noted that all included interactive games and sociodramatic activities embedded in each session have the ability to be adapted and tailored dependent on individual or group needs and preferences (ie, incrementally more challenging). All items and adaptations will be recorded for reporting purposes and to ensure reliability and the ability to replicate across groups. The PI (LS) will monitor the progress of the intervention delivery and programme documents each week for the trial duration.

### Fidelity monitoring

The intervention will be delivered according to the PEERS Plus manual. Clinicians leading the sessions are allied health professionals (ie, occupational therapists, speech pathologists, social workers and clinical/neuropsychologists) with experience conducting group and individual sessions in this practice area. Two clinicians per group will be required to deliver the intervention. Each group will have at least one clinician trained in the use of PEERS. Clinicians who will deliver the intervention (RG, NH and BT) have completed certified training to deliver PEERS for adolescents in its original formats.

Before intervention commences, the intervention manual will be shared with the clinicians to facilitate their preparation and delivery of the intervention. All procedures that have been developed will be clearly outlined and discussed in preintervention meetings. The content and dose of overall intervention components delivered will be documented and discussed during biweekly post-session meetings.

A checklist will be used to capture each child–caregivers dyad’s weekly attendance, participation, and completion of homework tasks.

### Procedures for screening

Eligibility screening and intake procedures will follow the PEERS for adolescents manual, which includes a 10–15 min telephone screen, followed by a screening interview (T0, 50 min in total held via Zoom or in-person pending caregiver–dyad preference) in which the child and their caregiver will be interviewed to learn more about the child’s social challenges and needs, determine the child’s motivation to participate in the programme towards developing friendships and describe the programme and commitment. All eligible child–caregiver dyads will attend baseline assessments in person (T1) and will be randomised to participate in either the social skills training immediately or waitlist usual care (refer to figure 1 for the outline of expected timeframes). Baseline assessments will be conducted by research clinicians at the Queensland Cerebral Palsy and Rehabilitation Research Centre at the Centre for Children’s Health Research in Brisbane, Australia. T1 baseline assessments are anticipated to take approximately 1 hour per participant to complete. This timeframe estimate is based on the cumulative total duration of all assessment measures.

### Screening and descriptive measures

Table 2 summarises the timeline of the assessment points of each of the measures throughout the trial, with detailed descriptions below.

**Table 2:** Screening and descriptive measures, outcome category/purpose, respondent and timepoint

Measure	Outcome category/purpose	Respondent	Timepoint
WASI-II	Screening for eligibility	C	T0
BRIEF (first edition)	Descriptive measure	P	T1
Conners third edition	Descriptive measure	P	T1
Demographic questionnaire	Descriptive measure	P	T1
COPM	Primary outcome	C, P	T1, T2, T3, T4, T5, T6
SSIS-SEL	Secondary outcome	C, P	T1, T2, T3, T4, T5, T6
QPQ	Secondary outcome	P	T1, T2, T3, T4, T5, T6
PECK	Secondary outcome	C	T1, T2, T3, T4, T5, T6
TCSSK	Secondary outcome	C	T1, T2, T3, T4, T5, T6
CSCQ for PEERS	Secondary outcome	P	T1, T2, T3, T4, T5, T6
Kidscreen-27	Secondary outcome	C, P	T1, T2, T3, T4, T5, T6
SDQ	Secondary outcome	P	T1, T2, T3, T4, T5, T6
CHU9D	Secondary outcome	P	T1, T2, T3, T4, T5, T6

BRIEF, Behaviour Rating Inventory of Executive Function; C, child; CHU9D, Child Health Utility 9D; COMP, Canadian Occupational Performance Measure; P, parent/caregiver; PECK, The Personal Experiences Checklist; PEERS®, Caregiver Social Coaching Questionnaire for PEERS®; QPQ, Quality of Play Questionnaire; SDQ, The Strengths and Difficulties Questionnaire; -SEL, Social Skills Improvement System Social-Emotional Learning Edition Rating Forms; TCSSK, The Test of Child Social Skills Knowledge; WASI-II, Wechsler Abbreviated Scale of Intelligence, second edition

### Initial screening

The WASI-II has evidence of acceptable validity and adequate reliability.^62^ A clinical neuropsychologist will administer the screener at the screening interview, where both verbal and non-verbal intellectual functioning will be measured. If a recent neuropsychology assessment has been completed (within 12 months prior through the Queensland Paediatric Rehabilitation Service), permission will be sought from the caregiver to share data to avoid unnecessary assessment burden.

Following this appointment, the Behaviour Rating Inventory of Executive Function (BRIEF),^65^ the Conners third edition^66^ and a demographic questionnaire will be completed at T1 to gain information on the cohort characteristics. The BRIEF is an 86-item parent-completed questionnaire that assesses executive function behaviours in the home environment,^65^ while the Conners third edition is a norm-referenced assessment tool used to obtain observations about the young person’s behaviour.^66^ A demographic questionnaire devised for the purpose of the study will be completed by the child’s caregiver. The demographic questionnaire aims to capture sociodemographic information pertaining to each child–caregiver dyad (eg, age and codiagnoses).

### Primary outcome

The Canadian Occupational Performance Measure (COPM)^51^ will be used to assess individual social participation goals over time. The COPM has high test–retest reliability (intraclass correlation coefficient, ICC 0.76–0.89) and is responsive to change. A change of two points or more is considered clinically meaningful.^51^ The COPM will be administered through a semistructured interview with child–caregiver dyads. The goals identified will focus on specific social skills that may assist participants in making and maintaining friendships, such as joining a conversation with a group at school, as well as social participation, like the frequency of get-togethers.

Each child–caregiver dyad will jointly identify 2–3 social goals. The current performance and satisfaction with performance will be rated jointly by child–caregiver dyads using a 10-point numerical rating scale. A rating of 1 indicates poor performance and low satisfaction, while a rating of 10 indicates excellent performance and high satisfaction. At each assessment point, baseline goals will be reassessed; any additional goals will not be formally documented or scored via the COPM. Child–caregiver dyads will receive a copy of their goals; however, they will not have access to the ratings for their goals.

### Secondary outcomes

The SSIS-SEL^52^ is a revised version of the Social Skills Rating System Rating Scales (SSIS-RS),^57^ one of the most routinely utilised questionnaire-based measure of children’s and youths’ social skills.^67^ The SSIS-SEL are a range of questionnaires (student, caregiver and teacher) assessing social–emotional learning skills (ie, social and self-awareness, relationship, responsible decision-making and self-management) in children and youth aged 3–18 years from multiple perspectives.^52^ Test–retest reliability (r=0.73–0.91) and internal consistency for all subscales is high (α=0.89–0.97), with total scale internal consistency estimates comparable between the SSIS-SEL and SSIS-RS.^52^ School-aged children will complete the self-report form, and the child’s primary caregiver will complete the caregiver version of the SSIS-SEL. SSIS-SEL Rating Forms (ie, student, caregiver and teacher) contain 46–51 items, completion time is approximately 10–20 min.

The QPQ by Frankel and Mintz (2011) is a 19-item parent-completed questionnaire that measures the frequency of hosted and invited get-togethers and quality of children’s play during recent playdates on a 4-point Likert scale.^68^ The scale has been utilised as an outcome measure in previous studies testing the efficacy of GSSIs and takes approximately 3 min to complete.^40 41 69^ The QPQ was created through a factor analysis of 175 young people and has a coefficient alpha of 0.87 for the conflict scale. A RCT of PEERS for adolescents revealed a Spearman correlation of 0.55 for the conflict scale and 0.99 for the frequency of hosted or invited get-togethers between parent and youth ratings at baseline (all p’s<0.001).^40^

PECK is a 32-item self-report dimensional assessment of a young person’s experience of being bullied.^54^ The tool has been featured in several evaluations of the effectiveness of bullying prevention programmes. It is suitable for young people aged 8–15 and takes approximately 10 min to complete.^67^ The PECK includes items for four subscales related to relational, physical, cultural and technology-based bullying. Young people are asked how often they have experienced each of the behaviours in the previous month. Responses are rated on a five-point Likert scale, ranging from ‘never’ to ‘most days’.^54^ The PECK has demonstrated good to excellent internal consistency for all four subscales (Cronbach’s alpha range=0.78–0.91; a=0.91 for verbal–relational bullying, a=0.90 for cyberbullying, a=0.91 for physical bullying and a=0.78 for bullying based on culture) and has adequate test–retest reliability (range r=0.61–0.86; r=0.75 for relational verbal bullying, r=0.86 for cyberbullying, r=0.61 for physical bullying, r=0.77 for bullying based on culture).^54^

The TCSSK is an adapted version of the Test of Adolescent Social Skills Knowledge-Revised (TASSK-R).^70^ The 22-item self-report questionnaire will measure a child’s knowledge of the specific skills taught throughout the PEERS Plus programme. The TCSSK takes approximately 5–10 min and requires participants to select the best option from two available answers. Total raw scores range from 0 to 22; a higher total score reflects higher knowledge of social skills. The TCSSK was adapted for use in this research project; accordingly, psychometric properties are not available.

The Caregiver Social Coaching Questionnaire for PEERS (CSCQ for PEERS) is a self-report questionnaire containing 10 items. The CSCQ takes approximately 3–5 min to complete and measures a caregiver’s knowledge, skills and confidence in coaching their child in various social situations and environments. Caregivers’ responses for the 10 items are rated on a Likert scale from 1 (strongly disagree) to 7 (strongly agree). The CSCQ for PEERS was developed for use in this study, psychometric properties have not been investigated.

The KIDSCREEN questionnaires are a series of instruments developed for surveying health-related quality of life (HRQoL) in young people between the ages of 8 and 18.^55^ KIDSCREEN-27 (short version) is a generic measure of HRQoL and has 27 items in five Rasch scaled dimensions: Peers and Social Support, School Environment, Physical Well-Being, Autonomy and Parents and Psychological Well-Being. The measure has shown good scale characteristics (eg, internal consistency; Cronbach’s alpha>70), convergent and criterion validity.^55^ Caregivers will complete the parent proxy version, and school-aged children will complete the self-report form. Estimated completion time is 10–15 min.

The SDQ is a 25-item behavioural screening questionnaire for young people aged 2–17.^56^ The SDQ is divided between five scales, including emotional symptoms, conduct problems, peer relationship problems, hyperactivity/inattention and prosocial behaviour and takes approximately 3–5 min to complete. Caregivers will complete the parent version of the SDQ, which demonstrates strong psychometric properties, including adequate test–retest reliability and internal consistency (*α=*0.80)*.*^71^

The CHU9D is a generic preference-based measure of HRQoL suitable for children and adolescents. The instrument takes approximately 5 min to complete and has nine dimensions (worried, sad, pain, tired, annoyed, schoolwork/homework, sleep, daily routine and able to join activities), each with a range of five possible responses (scored 1–5).^57^ The measure is appropriate for computing QALYs for young people aged 7–17 years; caregivers will complete the parent proxy version.

At the completion of the PEERS Plus programme, a qualitative interpretive description methodology will be employed to capture patterns and themes related to participants’ experiences and perceived acceptability and feasibility of the programme. Interviews will be conducted as focus groups with caregivers and children separately to explore the lived experience of primary and early high school delivered PEERS Plus. All participants will be invited to join the focus groups on programme completion. Semistructured interviews will occur immediately postintervention (ie, session 12) after the completion of all child and caregiver outcome measures/questionnaires to avoid bias. Two semistructured interview guides (ie, a 1 hour caregiver guide and a 20 min child guide) will be used across groups (ie, all immediate and waitlist groups). Interviews will be conducted in person by a researcher who is not involved in the intervention’s delivery, audiotaped and transcribed verbatim. Thematic coding of transcripts will be completed using NVivo software following analysis principles as outlined by Thorne (2016).^72^

### Process outcomes

The number of participants who: (1) are approached for this intervention, (2) commence the programme, (3) complete the programme or (4) decline the programme will be recorded. Reasons for non-participation will be sought and documented. An issues register will be developed to record any technical or logistical difficulties, participant, service delivery or organisational issues.

### Data management

Participants’ data, assessment and administration forms obtained for this research that could identify participants will be deidentified with a coded unique identification number and then stored in a locked filing cabinet at the Queensland Cerebral Palsy and Rehabilitation Research Centre. Data collected and/or uploaded to electronic formats for comparison and analysis will be managed by The University of Queensland through a secure online database.

Group video footage of each session will be transferred to the same secure database by a research team member after each session. Similarly, after the completion of focus group interviews, audio recordings will be uploaded to the same secure database. All information will be treated as confidential and securely stored, adhering to the University of Queensland institutional protocol and the Australian Code for the Responsible Conduct of Research. Final raw trial data will only be accessible to the study investigators or, on reasonable request, made available to other researchers and/or funding bodies, as needed, for the purposes of meta-analysis/systematic review and/or confirmation of statistical results. Identifiable data will not be made available unless separate ethics approval is sought. Aggregated data will be presented at conferences and submitted for publication.

In the event that a participant terminates their involvement in the trial, they will be provided the opportunity to complete outcome measures if applicable. All data will be deidentified prior to the dissemination of results. At the completion of the study, data will be archived as per the Children’s Health Queensland Research Ethics guidelines and institutional protocol.

### Sample size

A total of 32 participants will be recruited to pilot test the PEERS Plus programme. This number will allow for a minimum of 16 participants per group. This sample size will give us 80% power to detect a 2-point difference (clinically meaningful difference)^73^ on the primary outcome, the COPM, assuming a SD of 2^74^ with α=0.05 and buffering for 10% attrition.

### Management of withdrawals

Before consenting to participate, all participants will be made aware that they are free to withdraw from the study at any time without penalty. Participants who withdraw will not be replaced.

### Statistical analysis

Analyses will follow standard principles for RCTs, using two group comparisons on all participants on an intention-to-treat basis. Intention-to-treat analysis will be employed to reduce bias and ensure that all participants allocated to either the intervention (PEERS Plus) or control group are analysed together as representing that ‘treatment arm’ whether or not they received the intervention or completed the study.

Summary statistics will be calculated using means and SD or medians and IQR for continuous measures, depending on data distribution. Ordinal data will be summarised using counts and percentages. The approach to handling missing data will depend on its frequency and the underlying factors contributing to its incompleteness. For example, multiple imputation will be used to address missing data when it is believed to be missing at random. Statistical analysis will be conducted using Stata V.18, while qualitative data analysis will be performed using NVivo V.14.

The primary comparison H1 (attainment of social participation goals) immediately post the intervention at 12 weeks will be based on the COPM and will be between treatment groups using generalised linear models. Analyses will use similar methods to compare the outcomes between groups for secondary outcomes and timepoints (social skills and competence, quality of children’s play, the experience of being bullied and HRQoL).

This study will estimate the costs (both direct and indirect) associated with implementing the PEERS Plus programme. This study will adopt micro-costing/bottom-up approach to estimate the average costs of PEERS Plus groups. This costing study will be conducted from the Australian health system perspective, in which direct consumption of resources for providing the PEERS Plus will be measured. The cost analysis will involve a three-stage process: identifying resource use, measurement and valuation. The intervention cost will include consumables required for providing distinct therapy/interventions under the PEERS Plus and time spent by the trainers/therapists to provide in-person sessions to the adolescents and their caregivers. This information will be valuable to policymakers who need to allocate resources for this evidence-based programme.

### Patient and public involvement

The present protocol was developed after the completion of a qualitative study conducted with 27 adolescents with brain injury and 31 caregivers who participated in PEERS.^75^ This study has helped identify key considerations for future clinical and research use of PEERS with a population of young people with brain injuries.^75^

For the duration of the trial, we will convene biannual to quarterly meetings with an advisory group consisting of relevant stakeholders with lived experience of brain injury. The suggestions and recommendations raised in stakeholder meetings are anticipated to further assist in refining the protocol design, recruitment and engagement strategies, data interpretation and knowledge translation of the consequent study.

### Ethics and dissemination

This study has been approved by the Medical Research Ethics Committee of The University of Queensland (Project Number 2022/HE002031) and the Children’s Health Queensland Hospital and Health Service Human Research Ethics Committee (HREC/22/QCHQ/87450). This trial has been registered with the Australian and New Zealand Clinical Trial Registry (ACTRN12623000515695). Recruitment and participant informed consent processes will be completed in accordance with institutional ethical procedures. Children will only be included if written consent has been obtained from their guardian and if they have the capacity to provide some form of consent to participate (online supplemental material 1). Any significant protocol modifications will be made only after liaison with the research ethics boards, registered at Australian and New Zealand Clinical Trial Registry, and subsequently described in the subsequent publications. Dissemination plans include peer-review publication of study results, presentations and instructional workshops at national and international conferences.

Recruitment for this study commenced in May 2023 and will continue until late 2024. Participants will be followed up until 2025–2026.

## Discussion

This study details the protocol for a parallel waitlist pilot randomised two-group pretest post-test study investigating the efficacy of the PEERS Plus on improving social functioning for school-aged children with a brain injury. The study addresses a recognised gap in the continuum of care for primary and early high school-aged children with brain injury, and to the best of our knowledge, will be the first RCT to pilot an adapted version of PEERS in this population.

We hypothesise that participation in PEERS Plus will increase self-reported and caregiver-reported social participation and functioning compared with waitlist control. Additionally, we will explore participants’ experiences of participation in PEERS Plus and collect process-related outcomes, which will inform the implementation of the programme in future research and clinical practice if it is shown to be effective. Due to the nature of the PEERS Plus intervention, participants and the intervention providers will not be blinded to treatment allocation.

We anticipate that children with brain injuries who complete PEERS Plus will improve their social skills and social competence and develop skills to make and keep friends with an overarching positive impact on quality of life. Importantly, we anticipate that caregivers will develop the skills to coach their child in challenging social situations, and these skills will enable the sustainability of outcomes of this intervention in the longer term. Inclusion of a 3 and 9 month follow-up to assess maintenance of treatment gains aims to capture the sustainability of these outcomes.

A limitation of this study is that it excludes many children who may struggle with social functioning and participation, such as children with communication difficulties, intellectual disabilities and visual or auditory impairments, based on the inclusion criteria. Given the paucity of studies examining social skill interventions with children and young people with brain injury,^76^ we anticipate that the new evidence generated by this project will guide further investigation of social skills interventions for children with brain injuries and may provide emerging evidence for efficacy.

## supplementary material

10.1136/bmjopen-2024-095354 online supplemental file 1
