# Supplementary material for: Investigating a caregiver-assisted social skills group programme for primary and early high school-aged children with acquired brain injury or cerebral palsy: protocol for a pilot mixed-methods, two-group randomised trial of PEERS Plus
Source: BMJ Open. 2025 Jan 4;15(1):e095354. doi: 10.1136/bmjopen-2024-095354 (PMC11749881; doi:10.1136/bmjopen-2024-095354)
Supplement: online supplemental file 1 [file bmjopen-15-1-s001.pdf]

Children's Health Queensland Hospital and Health Service
Participant Consent Form (Parent/Guardian Version)

Participant ID Number: _______________

Project Title: A mixed methods randomised trial of the Program for the Education and Enrichment of Relational Skills (PEERS®) for primary school children with acquired brain injury and cerebral palsy.

HREC Reference number: [Insert number]

Parent/Guardian

I have read the above information. I have asked all of my questions and received answers. I agree to enroll in the following parts of this study with my child.

☐ The therapy and assessments

☐ The video recording of the group sessions for evaluation purposes

☐ The use of the video recording of the group sessions for future training and education of other researchers and health professionals

_______________________________________ ____________________

Signature Date

_______________________________________ _______________________________________

Print Name

CHIEF INVESTIGATOR

I have fully explained to the parent/guardian .... the nature and purpose of the program and the procedures to be employed as described above and such risks as are involved in their performance, and I have provided the parent/guardian with a copy of the Information Sheet.

______________________________________ ____________________

Signature of Investigator Date

______________________________________ ______________________________________

Print Name Position

INDEPENDENT WITNESS

I have witnessed the receipt of an Information Sheet by the parent/guardian and exchanging of information between the investigator and the parent/guardian about the study.

An auditor witness would optimally discuss the study with the subject and witness the subject signature

______________________________________ ____________________

Signature of Witness Date

______________________________________ ______________________________________

Print Name Position

IF DEEMED APPROPRIATE:

CHILD PARTICIPANT ASSENT

I have read the above information. I have asked all of my questions and received answers. I agree to take part in this study.

______________________________________ ____________________

Signature of Child/Adolescent Date

____

Print Name
