# Supplementary material for: Investigating a caregiver-assisted social skills group programme for primary and early high school-aged children with acquired brain injury or cerebral palsy: protocol for a pilot mixed-methods, two-group randomised trial of PEERS Plus
Source: BMJ Open. 2025 Jan 4;15(1):e095354. doi: 10.1136/bmjopen-2024-095354 (PMC11749881; doi:10.1136/bmjopen-2024-095354)
Supplement: Supplementary file 2 [file bmjopen-2024-095354.reviewer_comments.pdf]

PEER REVIEW HISTORY

BMJ Open publishes all reviews undertaken for accepted manuscripts. Reviewers are asked to complete a checklist review form and are provided with free text boxes to elaborate on their assessment. These free text comments are reproduced below.

ARTICLE DETAILS

Title (Provisional)

Investigating a caregiver-assisted social skills group program for primary and early high school-aged children with acquired brain injury or cerebral palsy: protocol for a pilot mixed-methods, two-group randomised trial of PEERS® Plus

Authors

Thompson, Bianca; Gilmore, Rose; Hilton, Nicola; Barfoot, Jacqui; Moody, Christine T; Whittingham, Koa; Keramat, Afroz; Boyd, Roslyn N; Sakzewski, Leanne

---

VERSION 1 - REVIEW

**Reviewer**	**1**
**Name**	**Wallen, Margaret**
**Affiliation**	**Australian Catholic University Faculty of Health Sciences, School of Allied Health**
**Date**	**03-Jan-2024**
**COI**	**I have no completing interests**

Thank you for the opportunity to review this proposal. This is an important topic, addressed by an acclaimed research group known for addressing the spectrum of issues confronting children with ABI and CP as well as their families, in thorough and rigorous ways.

This appears a carefully thought out study, which is clearly adhering to standards for completing RCTs. It is also really well written and a pleasure to read – free from errors and awkward expression.

I have approached the review in an attempt to provide feedback to address the rigour of reporting and also the kinds of information that will assist in inclusion in future systematic reviews/metanalyses, replicating the study and in interpreting/understanding the final study publication. I hope that drawing your attention to the issues I have outlined below will help you to craft a protocol for publication which gives full justice to this important and well designed study.

The final study will make a very important contribution to the field. It was great to see mention of the kinds of activities to ensure findings are disseminated and knowledge translated.

Abstract.

Word limit may be a problem, but the abstract does not address the nature and analysis of the qualitative arm of this study.

Introduction

This section of the manuscript is informative, it flows in a logical sequence, identifying the nature, extent and implications of the problem. It progresses to identify PEERS® as an evidence-based option to address the problem, including the evidence for involvement of caregivers.

Given the primacy of parent involvement (title, inclusion of parent groups) mention/discussion of why/how caregiver involvement is proposed to enhance outcomes is pivotal and needs inclusion.

Page 4/27, line 26: This is an awkward sentence and revising it would assist readers to readily understand your point. "While social functioning difficulties may be problematic during school years, if not addressed, it is likely they will persist into adulthood, placing individuals at risk for multiple forms of maladjustment problems19 and mental health problems". Suggest considering: Unaddressed social functioning difficulties during school years are likely to persist into adulthood, placing individuals at risk for multiple forms of maladjustment problems19 and mental health problems.

Page 4/27, line 53: Suggest deleting "recent" from the following sentence: Recent meta-analyses of GSSIs for young people with… Some are only relatively recent and it reduces duplication of the word in the next sentence.

Page 5/27, line 25: Please consider the terminology "treatment" in the following sentence and whether PEERS® can be considered a treatment. "As an evidence-based caregiver-assisted group social skills intervention, PEERS® is associated with social skills improvements and long-term maintenance of treatment gains in social competence 1–5 years following intervention." Please also reconsider use of the term "therapy" throughout.

Page 5/27, paragraph starting line 23: Given the central nature of caregiver assistance to this research, this paragraph needs to include/delineate the purpose and content of the caregiver concurrent program.

Page 5/27, paragraph starting line 42: It is important to report actual data for the existing evidence for PEERS® with children with ABI/CP – as you have responsibly done. The study you report, however, found no between group differences for the primary outcome. This is an important finding and rather than avoid declaring it, this study provides an opportunity to address this.

Pages 5 and 6/27 – no timepoints are mentioned in the aims and hypotheses. It seems the only time that the primary endpoint is mentioned is in the abstract. This should be addressed in the methods to set the reader up well for reading subsequent material.

What is PEERS® Plus? Can it be described briefly, as it does not have a context or a reference given when first introduced. Some of this will be clear in the methods, but just introduce the reader to it here.

Methods

Page 6/27, line 52: PEERS® is a very active intervention. Consider using alternative terminology to "receive PEERS® Plus" for example, "participate in PEERS® Plus".

A general point: This trial is described as a pragmatic trial, but also mentions exploring efficacy. How is this a pragmatic trial? Also, my understanding is that pragmatic trials can explore effectiveness, but not efficacy. Please consider this and address accordingly.

Page 6/27 under primary and secondary hypotheses – age limits are 8 to 13, which is different to the age group noted in Figure 1. Likewise age limit in recruitment section and inclusion criteria are different from Figure 1. Check all for consistency for school year also - years 3+ or 4-6.

Page 6/27 timeframes – mention 3 and 12 months follow up, but also a 9-month follow up is mentioned in Figure 1 and top of page 4/27. In contrast the abstract reports weeks and highly responsibly specifies weeks following baseline. This can all be brought into alignment to ensure that the task of reading and following endpoints is clear to a reader.

Page 7/27, paragraph starting line 12: It is responsible to use reporting standards to guide reporting of the RCT, protocol and intervention. Missing here, are guidelines/standards around reporting the qualitative aspect of this study, although this statement should be tempered according to the interesting viewpoints of this article https://journals.sagepub.com/doi/10.1177/1049732321994114

As I read on, the reporting of the focus groups is minimal. The title of the protocol is clear that the study is mixed methods, but the qualitative arm is underreported. More details are needed around the design and methods for the focus group, and analysis/interpretation of the qualitative data. A checklist may give guidance on the information which would complete the information for readers.

Recruitment

If recruitment commenced in May 2023, include details of ethics approvals in this protocol. I note it was included at the end of the paper. It would be helpful to reduce duplication.

Who is speaking with families and children about the study and what is their relationship to the researchers, interventionists and data assessors? Are children given the opportunity to provide their approval/consent/assent to be involved?

Include information on how participants were "invited to join the study", line 23-24, for example, phoned by a member of the study team, emailed etc.

Inclusion criteria

Briefly describe screening process mentioned page 7/27, line35

Line 39: explain source of IQ assessments.

Define what is meant by "basic proficiency in English" what do they need to be able to do, eg read participant information letters, understand the content of the sessions, etc.

Randomisation.

More detail is needed to assist with understanding the randomisation and allocation concealment processes. For example, is each consenting participant allocated a study number, so that after baseline assessment, the appropriate number is selected by study personnel. Are the envelopes available to the baseline assessor at time of assessment or is a phone call needed to an independent person.

Blinding

Please consider terminology in relation to COPM. It is an active semi-structured interview and use of the word "administered" does not capture its nature. Instead of "will be administered", consider "will be completed by child…"

It is important, as you have done, to declare inability to blind outcomes, due to their self-report nature. It is also important to report (and provide a rationale), as you have done, that outcomes assessors won't be blinded. However, COPM is self-report by child and family and therefore can't be blinded, regardless of whether the outcomes assessor is blinded to group allocation. This section needs to be altered to reflect that COPM is self report and therefore not possible to be blinded, and secondarily, the status of the assessors completing the COPM semistructured interviews

Are follow up COPM assessments completed blinded to previous scores?

PEERS® Plus Intervention section.

Clarify various terms used – therapist, interventionist, clinicians. It appears they refer to the same role - definition and use of the same terminology will reduce reader uncertainty.

Who adapted it PEERS® Plus, why, how was it adapted and how is it different to PEERS®. Does it have any relationship to the study mentioned at end of introduction by Gilmore et al.?

Procedures for screening

It appears that T0, T1 are not used consistently, eg in page 6/27 line 51 T0 appears to mean the baseline assessment but Page 10/27: T0 appears to indicate the screening process. Check and address (including Table 2 and Figure 1) as needed for consistency. Also check

endpoints for consistency between text and figure 1. It would be helpful to clearly establish, in the methods section, what the T0 through T6 noted in Figure 1 refer to.

Line 48: specify whether the baseline assessment is in person or can be online as per screening interview.

Initial Screening.

WASI-II:

Reference 3 does not appear to be an acceptable primary source of information about the reliability and validity of this tool.

The WASI-II is to be completed at the screening interview which can be via Zoom or in person (page 10/27 line 43). Can the WASI-II be completed remotely?

The purpose of the WASI-II is implicit as it ties directly to an inclusion criteria. Now tie the BRIEF and Conners to the inclusion criteria, including the cut offs for eligibility.

Primary outcome COPM

As the primary outcome and as a study protocol, more information is needed – here are some suggestions which I hope will help fully inform the reader about the rigour of this assessment process. How is it completed, ie will you use a semi structured interview to identify social participation goals? How will it be adapted to achieve social participation goals rather than the self care, productivity and leisure goals usually derived from the COPM? Who will complete it – the child and/or parent and how will this be determined? What scores are generated, eg average of 2 to 3 goals on a 1-10 point scale, specify the direction of the scale. Specify that performance and satisfaction with performance will be rated. What happens at each endpoint eg just the re-rating of the baseline goals, or additional goals. Will respondents have access to original ratings for goals or will they be blinded to original ratings?

Further, please check the source for the change of two points – usually the two points is considered to be clinically important difference rather than statistically significant. The following link may also be helpful. https://www.thecopm.ca/casestudy/responsiveness-of-the-copm/

Secondary outcome measures. Important information is included for these outcomes, but not consistently for each measure. Please address each issue for each measure (including COPM), and where information is not available, specify that this is the case.

• Evidence of reliability, validity and responsiveness to change. The latter, in the context of social skills programs is critical. Specify where evidence doesn't exist for these psychometric properties

• Age of child that the respective child or proxy report questionnaire has been validated (used) with

• Domains covered (if applicable)

• Who completes it

• Number of items

• How rated (e.g., 4 point Likert)

• Scores derived and used for analysis, range/magnitude of the scale (eg 1-10 point scale) and direction of better function/improvement

• Time taken to complete

• Minimally clinically important difference or other indicator of clinical importance.

Provide estimation of the time to complete all measures for child and family and any evidence for the likelihood of this being achievable.

Further information needing to be included: where are the assessments carried out, who by, what is their relationship to interventionists and research team?

Qualitative aspect of study

Page 12/27, paragraph starting line 44. More details on the methods for the qualitative aspect of this study are needed, how many focus groups, who is eligible, how long are the focus groups, are they face to face or online, provide or describe the focus group guides, how are they adapted for each focus group etc. How long after completion of intervention will the focus groups take place, will both groups have the opportunity to participate in a focus group etc. More detail on the approach to data analysis would strengthen the reporting of this aspect of the study also.

Page 13/27: Data management

This section would benefit by revisiting and tightening. Conflicting information is included about when data are deidentified.

In relation to the following: “Final trial data will only be made accessible to the study investigators.” – specify this means raw data, as aggregated data will presumably be published and presented in conferences. Later on page 16/27, I note that you will make the data available to others, so this needs to be reflected in this section also, or at least made consistent.

What is done with the recordings of each session, ie what is the purpose of recording each session, this is not covered in the methods and should be.

What is done with the (audio and/or video) recorded focus group data and transcripts in terms of data management?

Statistical analysis

This section is brief, but it is responsible to specify an intention to treat approach.

How will missing data be managed?

What data (point estimate/variability) will be reported – I am thinking here about future inclusion of the study in metaanalyses.

It is preferable to present the analysis of the primary outcome before cost analyses. Also address the analysis of the primary and various endpoints, which differ for each arm of the study.

The inclusion of analyses around costs are not pre-empted as a study aim or in the methods section – this will need to be addressed adequately prior to inclusion in the data analysis section.

Public and Patient Involvement.

It is responsible to see the inclusion of an advisory group. This section would benefit by clarity around concrete contributions this group will make to the study – given the study is already underway. What do you plan to discuss with the group, for instance, any roadblocks, interpretation of any data, retention, development of focus group guides, knowledge translation, other?

Discussion

Is there a way of acknowledging that many children for whom social participation will be a substantial struggle are excluded from participating in the study (and from involvement with PEERS) - those with communication difficulties, ID, vision/auditory difficulties. How might this be addressed in the future?

**Reviewer**	**2**
**Name**	**Babaei, Meisam**
**Affiliation**	**North Khorasan University of Medical Sciences**
**Date**	**27-Mar-2024**
**COI**	**there isn't any competing interest with this review**

Hi Dear Author,

Discussion part is too short, please explain more.

VERSION 2 - REVIEW

**Reviewer**	**1**

Name **Wallen, Margaret**

Affiliation **Australian Catholic University Faculty of Health Sciences, School of Allied Health**

Date **24-Nov-2024**

COI

The authors have addressed the feedback and suggestions thoroughly and carefully.

VERSION 2 - AUTHOR RESPONSE

**Title:** Investigating a caregiver-assisted social skills group program for primary and early high school-aged children with acquired brain injury or cerebral palsy: protocol for a pilot mixed-methods, two-group randomised trial of the PEERS® Plus			
Comment Number	Comment	Author's response	Change made
**Reviewer 1:**			
1	The authors have addressed the feedback and suggestions thoroughly and carefully.		
**Reviewer 2:**			
1	N/A		
